# Development of an eHealth System to Capture and Analyze Patient Sensor and Self-Report Data: Mixed-Methods Assessment of Potential Applications to Improve Cancer Care Delivery

**DOI:** 10.2196/medinform.9525

**Published:** 2018-10-22

**Authors:** Alexander R Lucas, Michael B Bass, Nan E Rothrock, Mary L O'Connor, Mia R Sorkin, Jason Nawyn, Fahd Albinali, Lynne I Wagner

**Affiliations:** 1 Department of Social Sciences and Health Policy Wake Forest School of Medicine Winston-Salem, NC United States; 2 Feinberg School of Medicine Northwestern University Chicago, IL United States; 3 Massachusetts Institute of Technology Media Lab School of Architecture + Planning Massachusetts Institute of Technology Cambridge, MA United States; 4 QMedic Cambridge, MA United States; 5 Comprehensive Cancer Center Wake Forest Baptist Health Winston-Salem, NC United States

**Keywords:** cancer, care delivery, decision support, eHealth, mobile phone, survivorship, symptom monitoring

## Abstract

**Background:**

Capturing and Analyzing Sensor and Self-Report Data for Clinicians and Researchers (COMPASS) is an electronic health (eHealth) platform designed to improve cancer care delivery through passive monitoring of patients’ health status and delivering customizable reports to clinicians. Based on data from sensors and context-driven administration of patient-reported outcome (PRO) measures, key indices of patients’ functional status can be collected between regular clinic visits, supporting clinicians in the delivery of patient care.

**Objective:**

The first phase of this project aimed to systematically collect input from oncology providers and patients on potential clinical applications for COMPASS to refine the system.

**Methods:**

Ten clinicians representing various oncology specialties and disciplines completed semi-structured interviews designed to solicit clinician input on how COMPASS can best support clinical care delivery. Three cancer patients tested a prototype of COMPASS for 7 days and provided feedback. Interview data were tabulated using thematic content analysis to identify the most clinically relevant objective and PRO domains.

**Results:**

Thematic content analysis revealed that clinicians were most interested in monitoring vital statistics, symptoms, and functional status, including the physical activity level (n=9), weight (n=5), fatigue (n=9), sleep quality (n=8), and anxiety (n=7). Patients (2 in active treatment and 1 in remission) reported that they would use such a device, were enthusiastic about their clinicians monitoring their health status, especially the tracking of symptoms, and felt knowing their clinicians were monitoring and reviewing their health status provided valuable reassurance. Patients would, however, like to provide some context to their data.

**Conclusions:**

Clinicians and patients both articulated potential benefits of the COMPASS system in improving cancer care. From a clinician standpoint, data need to be easily interpretable and actionable. The fact that patients and clinicians both see potential value in eHealth systems suggests wider adoption and utilization could prove to be a useful tool for improving care delivery.

## Introduction

In 2014, there were an estimated 14.5 million cancer survivors in the United States, and this number is expected to reach 19 million in 2024 [1]. The aging population, increased rates of screening [2,3], and improved availability and quality of treatments have resulted in cancer survivors living longer [4]; over the next decade, the proportion of 5-year cancer survivors is expected to increase by approximately 37% [5]. The provision of high-quality medical care for this growing segment of the population has been identified by the American Society of Clinical Oncology as a priority [6]. Specific priorities include (1) monitoring of patient-reported outcomes (PROs) such as symptoms, health status, and quality of life; (2) adherence to treatment regimens; and (3) monitoring of lifestyle and health-protective behaviors [7-10].

Current evidence shows that collecting PROs including symptoms (pain, fatigue, and nausea), psychosocial well-being, and quality of life yields better clinical outcomes [11,12], including potential survival benefit [13]. Worsening symptoms might signal disease recurrence or progression or the need for medication or dosage adjustments. However, there is a great deal of variability in how PROs are actually being collected and implemented in oncology care [14]. Typically, this type of information is only gathered at routine clinic visits, if at all, and, therefore, may not accurately reflect day-to-day functioning. Furthermore, as patients do not want to burden their care team, they tend not to report symptoms unless specifically asked or they will wait for their next scheduled clinic visit to report concerns [15]. The systematic integration of PROs into clinical care through utilizing an electronic platform that offers the ability to communicate with patients in real time could lead to shorter response times, better symptom management, and ultimately better outcomes [16]. Furthermore, efforts to integrate precise and robust symptom measures such as Patient-Reported Outcomes Measurement Information System (PROMIS) have emerged over the last few years [17].

Adherence to medication has emerged as a particular concern because of the rise in the use of oral anticancer drugs [18]. In terms of lifestyle and health-protective behaviors, physical activity, time spent in sedentary pursuits (television watching), and diet or nutrition markedly impact the physical, functional, and psychological health status of patients and survivors [19-21]. Physical activity is an especially important indicator of physical functioning, health-related quality of life, risk for a decline in health status [22,23], and mortality outcomes [24], meaning its maintenance is an important clinical goal. Failure to manage these aspects of care can lead to increased risk of developing comorbidities and, therefore, to excess economic burden associated with medical care, time off work, lost productivity at home, and additional medical visits [25,26].

However, there are challenges to monitoring the health status of patients in-between clinic visits, soliciting clinically relevant data from patients and family members in an efficient manner, corroborating patient self-report data (eg, physical activity), and integrating multiple sources of clinically relevant data in the context of busy oncology practice. Addressing these challenges can be a daunting task, chiefly because clinicians and their staff are already plagued by numerous competing demands. Therefore, developing innovative ways for clinicians to monitor their patients’ behaviors and when needed providing guidance to help them with adherence to medicines, adoption and maintenance of healthy lifestyles, and cope with stress could potentially enhance the overall quality of supportive care and reduce the burden on both patients and their care teams. Electronic health (eHealth) models of care that leverage electronic health records, as well as digital and wearable technologies, are now emerging as an innovative strategy to reduce unmet care needs and support regular monitoring and interaction with patients between scheduled clinic visits [27].

Capturing and Analyzing Sensor and Self-Report Data for Clinicians and Researchers (COMPASS) is a device-agnostic eHealth technology platform that can passively and remotely monitor multiple domains of function and PROs. The COMPASS system includes (1) a device worn by patients to passively monitor physiological function; (2) an interface to sync with patients’ smartphone; and (3) a Web-based clinician interface to deliver customizable reports. The purpose of this study was to explore the user requirements for such a system to ensure that it can adequately support the breadth and range of functionality typically requested by practitioners in the field. A user needs assessment was conducted to establish design and use metrics of a prototype COMPASS system before conducting more comprehensive testing and evaluation in a larger-scale phase II study.

## Methods

### Participants and Procedures

This study was conducted at the Robert H Lurie Comprehensive Cancer Center (RHLCCC) of Northwestern University and was approved by its Institutional Review Board. Potential participants, including clinicians and patients, provided written informed consent prior to participation. Clinicians were provided with a description of the COMPASS system 7 days prior to the interview with instructions to think about potential benefits of the system. Patients were provided with a prototype of the COMPASS app and wearable sensors (Mio Alpha Sports Watch) for 7 days. Both clinicians and patients then completed a semistructured interview with a trained interviewer. All interviews were audiorecorded and transcribed.

### Clinician Interviews

Clinicians were eligible if they were current oncology providers at RHLCCC. A purposive sampling strategy was used to recruit a diverse sample of 10 oncology providers with regard to specialty and clinical practice foci. Clinicians agreeing to participate provided written, informed consent. We provided clinician participants with a description of COMPASS approximately 1 week prior to the interview and asked them to think about how they would utilize the system to inform clinical care delivery (Multimedia Appendix 1, “Thought Exercise”). Clinicians then completed in-person semistructured interviews with a trained interviewer. Software developers who designed COMPASS (FA and JN) participated in the interviews via conference call. Each interview lasted approximately 1 hour. Interview content included information about their clinical practice (eg, specialty and types of patients typically seen), metrics most pertinent to treatment decision making and goals of care, how COMPASS could help to inform clinical visits, and preferences for how patient data collected through COMPASS are summarized and presented. Multimedia Appendix 2 provides examples of clinician interviews.

Textbox 1 presents examples of questions posed to clinicians. The semistructured interview solicited preferences for the types of possible wearable sensors, PROs most relevant to clinical practice, preferences for the format of data visualization options, and communication preferences including the sharing of patients’ data and the frequency of contact with patients as a way of improving the delivery of care. The interview template evolved as interviews were conducted; therefore, only a subset of providers was asked to discuss the sharing of data with patients. Multimedia Appendix 3 provides a list of potential sensors that could be incorporated into the COMPASS system. Clinicians were provided with this list during the interviews to inform them of potential metrics that could be captured.

### Patient Interviews and Testing

To be eligible for participation, patients had to be aged ≥18 years, diagnosed with cancer (any type, all stages), and had to own a smartphone. Patients could be at any stage of treatment, including posttreatment. Patients provided written informed consent prior to participating in any research activities. Patients were identified through participating clinicians and were approached in a clinic regarding participation. In addition, patients were recruited through a study brochure and flyers placed in RHLCCC clinical practice areas and through outreach on social media sites such as Twitter and Facebook. Multimedia Appendix 2 provides examples of patient interviews.

**Figure 1:**
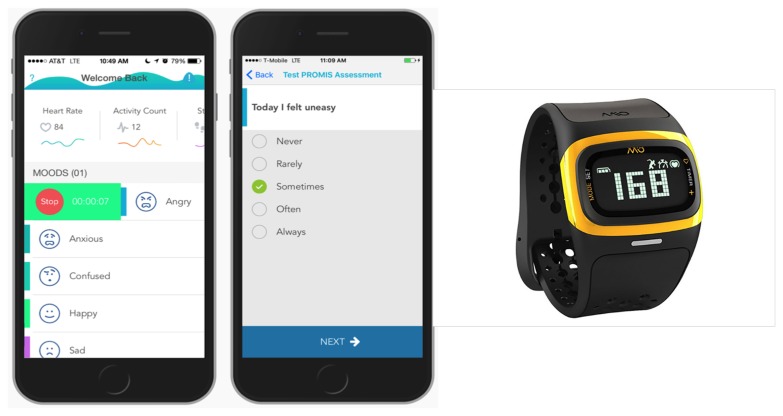
Screenshots of smartphone app (QMedic Health, Inc) and Mio ALPHA 2 (Mio Global) wristwatch device.

Each patient participated in two study visits. The first was conducted in-person at RHLCCC. Demographics and disease-specific information were gathered. A commercially available smartphone, with an armband (should they prefer to wear the smartphone rather than carry it), and Mio Alpha heart rate monitor wristwatch were provided to participants for the duration of study participation (7 days). The correct use of the technology was demonstrated, and participants were instructed to wear and interact with the wristwatch and smartphone app for 1 week. We asked participants to respond to brief PRO measures, with the explanation that data collected from smartphone-based surveys would not be assessed for content but rather for evaluating the feasibility and usability of this feature. Following the 7-day testing period, the second study visit was conducted at which time the devices were returned; the second study visit included a follow-up semistructured interview to collect data on wearability or usability of the device and patient experiences communicating with health care providers regarding data collected using the COMPASS system. The interview was approximately 1-hour long and was audiorecorded and transcribed. At the conclusion of the interview, a US $50 gift card was provided. Examples of patient interview questions are provided in Textbox 1. Figure 1 provides screenshots of the smartphone app and wristwatch device.

### Analyses

All interviews were subjected to thematic content analysis (TCA) [28,29]. For clinician interviews, TCA was used to organize data according to the frequency and, therefore, the relative importance of the responses. The TCA was completed by two independent coders to tabulate the most common symptoms, clinical concerns, potential applications of the system and themes. Any coding discrepancies were discussed and resolved by the senior author (LIW). In addition, a list was generated and a frequency for each topic recorded. For patient interviews, a conventional qualitative content analysis [30] was used to analyze responses. Transcripts were read several times by the analyst, who determined a coding scheme inductively. Transcripts were coded in ATLAS.ti 8.0 (ATLAS.ti Scientific Software Development GmbH, Berlin, Germany). After coding, segments of text were abstracted by code, reviewed for themes, and summarized.

**Examples of clinician and patient questions.**

**Clinician Questions**

- What would you want this system to measure and how?
- Which patient populations might benefit the most from Capturing and Analyzing Sensor and Self-Report Data for Clinicians and Researchers (COMPASS)?
- What do you perceive as the benefits of COMPASS?
- How would you like collected data to be presented or reported?

**Patient Questions**

- What was your experience of wearing the device?
- What is the frequency of conversations with your care team and what do you discuss?
- Some people find it difficult to keep track of certain things about their health in order to talk about them at a doctor’s visit. Do you ever find that’s true for you? What would make that easier?
- Is there anything you would not be comfortable with your medical team monitoring?

## Results

### Clinician Interviews

We enrolled 10 of 17 clinicians who were invited to participate (59% participation rate), including 5 physicians, 3 nurse practitioners, 1 clinical psychologist, and 1 physical therapist. Table 1 presents the details on clinicians’ characteristics and responses by specialty area and COMPASS features.

### Priority Areas for COMPASS to Assess

**Figure 2:**
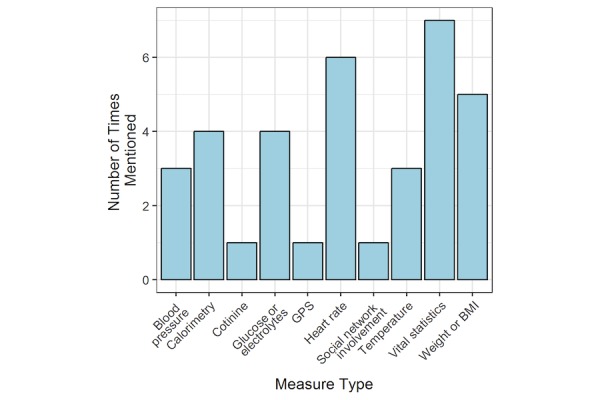
Objective measures. GPS: global positioning system; BMI: body mass index.

**Figure 3:**
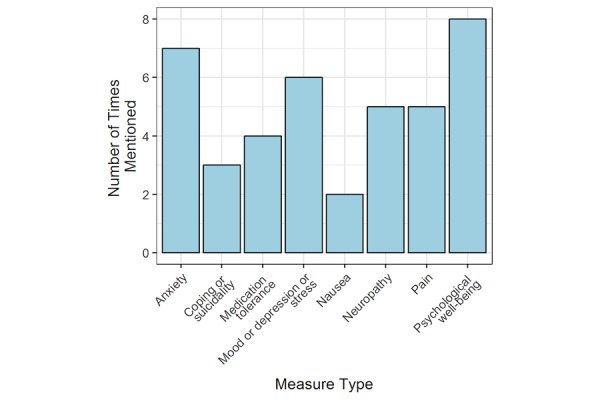
Patient-reported outcomes.

**Figure 4:**
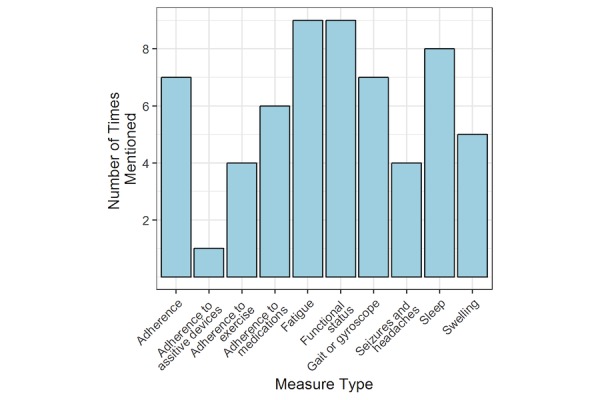
Content measured by both objective sensors and patient-reported outcomes.

Figures 2-4 present frequency distributions for content to assess outside of clinic visits. The objective measures that were deemed most valuable and relevant for informing clinical care were general vital statistics (n=7), heart rate (n=6), weight or body mass index (n=5), caloric expenditure (n=5), and glucose or electrolyte monitoring (n=4). When clinicians were asked about the use of a global positioning system or a monitoring system to track movements inside and outside the home, clinicians reported concern that patients would experience this as an invasion of privacy, and global positioning system data would not necessarily yield actionable results. However, data on general physical activity obtained from accelerometers or pedometers were considered potentially more useful. Domains that could be measured using PROs that clinicians were most interested in routinely assessing included psychological well-being (n=8), anxiety (n=7), mood, depression, and stress (n=6), pain and neuropathy (n=5), medication tolerance (n=4), and nausea (n=2). Figure 3 reflects an interest in types of data that could be captured by a combination of both wearable sensors and PROs. Functional status (including physical activity) and fatigue were mentioned by most clinicians (n=9), with sleep (n=8), gait (n=7), and adherence to treatment in general (n=7) the next most commonly reported. Adherence to medication (n=6) and lymphedema or swelling (n=5) was identified by roughly half of the clinician respondents.

Notably, an overall reflection from most clinicians was the need for data collected to be actionable. The following five main themes emerged representing data that were useful and actionable: (1) *Functional status (physical and cognitive)*, including information about daily physical activity, mobility constraints (ability to walk, balance, and engage in activities of daily living), weakness, cognitive abilities (forgetfulness), sleep (sleep-wake cycles and naps) and capacity for independent self-care; (2) *Indicators of disease progression (ie, health deterioration)*, including falls, seizures, and declining level of physical activity; (3) *Symptoms from disease (particularly disease progression) or treatment*, including side effects such as headaches, nausea, and increased pain from rehabilitation; (4) *Psychological well-being*, including anxiety, depression, and fear of recurrence; and (5) *Adherence to treatment and health behaviors*, including medication adherence, alcohol reduction, smoking cessation, and engaging in physical activity. In addition, clinicians discussed *test and imaging results* as important for evaluating response to treatment and determining ongoing treatment plans.

### Patients Most Appropriate for COMPASS

Clinicians’ perspectives on patients who could benefit the most from COMPASS differed by specialty. For example, all 3 rehabilitation clinicians felt that patients with functional limitations and who were at highest risk for events such as falls would benefit most from a system like COMPASS. For example, patients being treated for brain and spinal cord tumors were considered high risk for falls because of significant functional limitations secondary to disease. Other distinct groups that rehabilitation specialists felt could benefit from COMPASS were survivors, no longer in active treatment and who no longer were actively engaged in health care services, patients with pain (focusing on these patients during treatment could help to offset problems down the line), and older, overweight, and sedentary patients who typically have more comorbidities (eg, diabetes), and who were, therefore, also at greater risk for falls and frailty-related declines in function. Conversely, other clinicians indicated that younger patients who were more impacted by their diagnosis and who would also spend longer in survivorship were more likely to benefit from a system like COMPASS.

In addition, clinicians identified a separate patient group at risk and who could benefit from COMPASS—elderly men with a substance abuse history and poor social support. Other psychosocial concerns were for patient groups like breast cancer survivors with large treatment burden that could lead to sleep issues and depression. Three clinicians specifically talked about patients (and caregivers) who experienced high anxiety being able to communicate regularly via COMPASS. Finally, patients who had difficulty with treatment adherence could benefit from a system that could incorporate reminders, for example, those who had complicated treatment regimens and who could, therefore, be helped with structured reminders and regular check-ins.

### Summarizing Patient Data Collected Through COMPASS

Table 1 reports how clinicians of different specialties felt data should be presented and which platforms would make this most accessible. Overall, 5 clinicians wanted to see charts and graphs supported by qualitative written information to aid in interpretation, while other respondents wanted only charts and graphs or only written information. Owing to concerns about data overload, it was suggested that a summary of data with an option to expand to a more detailed view would offer the best usability. Data should only be provided when something abnormal was indicated.

**Table 1:** Clinicians’ characteristics and responses by specialty area and Capturing and Analyzing Sensor and Self-Report Data for Clinicians and Researchers (COMPASS) features.

Specialty area		Main concerns for patients	Uses for COMPASS	Format of data presented	Platform for viewing data	Sharing patient data
**Neuro-oncology**						
	Physician 1	Current rehab activity or need for referrals	Triggering alerts	Graphs or charts and qualitative data	Desktop personal computer (PC)	No
	Physician 2	Monitoring adverse effects of medications or interventions	Triggering alerts and triggering electronic interventions (eInterventions; eg, reminders)	Graphs or charts and qualitative data	Desktop PC	Yes
	Nurse practitioner	Current rehab activity or need for referrals and monitoring adverse effects of medications or interventions	Triggering alerts and eInterventions	Graphs or charts and qualitative data	Desktop PC	Yes
**Rehabilitation**						
	Physiatrist 1	Monitoring adverse effects of medications, tracking physical activity, and identifying comorbidity	Triggering eInterventions	Graphs or charts and qualitative data	Desktop PC and laptop	Yes
	Physiatrist 2	Nothing mentioned	Triggering alerts and eInterventions	Graphs or charts only	Laptop and tablet	Yes
	Physical therapist	Patient vitals for the safety of exercise	Triggering alerts and eInterventions and data summary at the point of care	Graphs or charts and qualitative data	No preference	Yes
**Cancer survivorship**						
	Physician (gastrointestinal cancers)	Medication adherence	Data summary at the point of care	Graphs or charts	Device agnostic (all)	Yes
	Nurse practitioner (breast cancer)	Monitoring adverse effects of medications, identifying late effects, and addressing nutrition concerns	Triggering alerts	Graphs or charts and qualitative data	Desktop PC	Yes
Surgical oncology nurse practitioner (gastrointestinal cancers)		Determining the need for referrals	Triggering alerts	Graphs or charts	Desktop PC	Yes
Supportive oncology clinical psychologist (general oncology, head and neck cancer)		Tracking lifestyle behaviors (smoking, alcohol, and physical activity)	Triggering alerts and eInterventions and patient networking	Graphs or charts	No preference	Yes

Five clinicians preferred to view data on a desktop computer as they reportedly felt it was more secure and had a large area for viewing data; 4 of 10 reported they were comfortable on all platforms. However, some clinicians (n=3) did not like to look at important data on the phone, and 2 clinicians expressed concerns that having data delivered to their personal phones might remove an important barrier between themselves and their patients. All but 1 of the clinicians were interested in the data being sharable between those who were providing care, reflecting that this may be the most useful part of the entire system, especially because cancer care is so multidisciplinary. Conversely, another clinician expressed that time is a valuable and limited resource and having a care plan where one person brings the data together to decide a course of action may be better than the raw data being viewed by multiple people, all of whom are reviewing and, perhaps, deciding a duplicative plan.

### Sharing of Data With Patients

A subset of 6 clinicians was asked whether they thought patients should be provided with their own data. Most clinicians (n=5) were in favor of seeing a data summary being provided. Reasons given were that it would help to engage patients with the technology and, perhaps, lead to better long-term use and could show patients their patterns. It was suggested by one clinician, who was in favor of data sharing, that, perhaps, some data should be presented in the clinic rather than via COMPASS, as this could allow the clinician to frame the information appropriately. For example, data related to imaging or other clinical interpretation should not be available outside the clinic where it could not be explained properly, and patients may become more anxious if they do not understand the data. In this study, 9 clinicians were asked about the utility of patients being able to annotate their data to provide context, and all agreed this would be valuable; however, they would still call or contact patients for clarification anyway. Furthermore, 3 of the clinicians felt that while valuable, this should only be optional and not required as it may increase the patients’ burden.

### Patient Interviews

We enrolled 3 patients, 2 of whom were diagnosed with brain cancer (one receiving active treatment and one 2 year posttreatment) and 1 had a previous diagnosis of lung cancer and was also 2-year posttreatment (Table 2). Owing to the variety of recruitment methods utilized, including social media, flyers in clinics, and on RHLCCC notice boards, we could not track a participation rate for patients.

### Wearability

Patients wore the Mio wristwatch devices for an average of 7 days prior to their interviews. Patients found the watch-like style of the device acceptable as it was generally comfortable, as long as it was not worn too tightly. However, 1 of 3 patients seemed somewhat uncomfortable. When asked if potentially wearing a tracking device like the Mio on a chain around the neck so it was close to the chest rather than on the wrist or whether they would prefer to wear the smartphone on an arm strap, 2 of 3 patients commented that these options were not favored. Patient 3 specifically responded that he or she would not want to wear a cell phone while running saying “I think it would be difficult, so no.” While not considered a particular theme, 1 patient also made comments relating to specific concerns about privacy, professional life, and intimacy. As part of wearability, patients also specifically noted the battery life of the wrist device. While Patient 1 was able to charge the device and iPhone nightly, Patients 2 and 3 had some trouble. Both patients reported a short battery life that was inconvenient.

### Data Capture

All patients reported times when they were unsure their data were being captured; this was either because the device was not positioned correctly or because the device and cell phone were not communicating. In addition, patients were asked to enter basic data on the dietary intake, mood, and activity. A small number of options were available in each category and patients reported that this task was easy.

### Tracking of Symptoms and Other Items

The tracking of symptoms during treatment and recovery was a particular area of interest for patients. Patients agreed that tracking a symptom was helpful; however, the symptoms experienced and preferences for tracking these symptoms varied by patient. Table 3 shows the kinds of symptoms that patients reported experiencing during their treatment, and Table 4 shows that in addition to tracking symptoms, patients also felt it would be useful to track other items.

All patients felt it would be useful to track their moods or feelings on an app during treatment to discuss with providers. One patient, in particular, felt this was important with regards to medications that may need adjustment. In addition, all patients reflected that having the ability to add freeform text to responses they had provided was important for linking activities or events with mood, changes in heart rate or stress, providing context to their data.

### Suggestions for Further Development of the System

Finally, patients reflected about preferences and suggestions for improvements for COMPASS; these included suggestions for improving the PRO descriptions and adding functionality to the surveys depending on responses given. For example, the device could provide guided relaxation exercises and imagery if they reported high levels of anxiety. The contact frequency should also be limited. One patient stated, “15 notifications in a row is unnecessary.” All patients wanted to be able to review their personal data, as they felt this would be “motivating.” In addition, the system could provide better support for reminders, including for taking medicines, filling prescriptions, and upcoming appointments. There were mixed responses to questions about the capacity of the device to contact significant others if there were concerns about their health.

**Table 2:** Patient characteristics and device preferences.

Characteristic	Patient 1	Patient 2	Patient 3
Cancer site	Brain	Brain	Lung
Treatment status	Current chemotherapy	2-year posttreatment	2-year posttreatment
Platform or device currently using	iPhone, laptop	Android	Personal computer, Mac laptop, Android
Platform or device preferred	iPad	Desktop and mobile	iPhone

**Table 3:** Patients’ symptoms.

Symptoms	Patient 1	Patient 2	Patient 3
Anxiety	✓		✓
Confusion	✓		
Constipation	✓		
Feeling sick		✓	
Fevers			✓
Headaches		✓	
Memory loss	✓		
Neuropathy	✓		
Pain	✓		✓
Respiratory infection			✓
Seizures or auras		✓	
Vision loss (peripheral)	✓		
Weakness	✓		
Weight loss	✓		

**Table 4:** Items to track.

Item to Track	Patient 1	Patient 2	Patient 3
Appointments		✓	
Confusion	✓		
Dietary intake or nutrition	✓	✓	
Exercise or activity, including heart rate and steps	✓	✓	✓
Fatigue or exhaustion	✓		
Fevers			✓
Headaches	✓	✓	
Medication use or prescriptions		✓	✓
Mood, including anxiety	✓	✓	✓
Pain	✓		✓
Progress	✓		
Seizures or auras		✓	
Weight		✓	

### Design Impacts of User Needs Assessment

The design of the prototype system represents an attempt to accommodate the most frequently requested features from the clinician assessment while minimizing the user burden on the patient population. Because a goal of this phase I study was to evaluate the feasibility of a system that integrates across multiple devices and data sources, efforts were made to realistically reflect the type of activities required to operate and maintain such a system. Specifically, the type and position of the wearable device were necessitated by technical constraints related to physiological sensing and capturing physical activity. Similar considerations were taken to provide a user interface on the mobile phone that accurately reflects that number, type, and duration of interactions required to collect the types of PROs most frequently requested by clinicians. Subsequent design iterations over both the clinician and patient interfaces should incorporate feedback on utility and usability from participants until an optimal balance between these objectives is achieved.

## Discussion

### Principal Findings

This study yields important insights regarding the initial feasibility and priority domains to inform the development of COMPASS, an eHealth platform designed to facilitate the patient-provider communication and improve supportive care outcomes in the cancer care setting. Clinicians were most interested in measuring and monitoring general vital statistics (heart rate, body weight, caloric expenditure, and glucose levels), functional status, symptoms (mood, depression, anxiety, and pain), and medication adherence. Importantly, measures needed to be actionable and integrate both objective metrics and PROs together to provide the richest and most clinically relevant understanding of the patients’ status. This was echoed by patients, who also wanted to be able to provide context to their data and responses. Patients were most interested in monitoring their symptoms, including pain, headaches, mood, and anxiety. In addition, they wanted to be able to track physical activity, diet or nutrition, medications, and appointments. Overall, patients who may benefit the most from a system like COMPASS depended on the clinicians’ specialty. Particular groups mentioned were the elderly, those with comorbidities such as diabetes, and those with complicated treatment regimens (oral chemotherapy).

The results of this study indicate that both clinicians and patients felt that a system like COMPASS had potential benefits for the delivery of cancer care. Given the shared interest and importance of symptom monitoring by both clinicians and patients, finding ways to improve this aspect of patient care seems critical. Recent studies have suggested that oncologists are often not sufficiently aware of their patients’ symptoms [31,32] but that when prompted, patients willingly provide this information [33]. However, integrating such self-report systems into routine clinical care with minimal disruption is of key importance [11], which is where eHealth-based platforms may offer significant potential. A recent qualitative needs assessment among 30 head and neck and breast cancer survivors supported these findings, revealing that survivors often felt their symptoms remained unknown to care providers [34]. In addition, they reported that the advantage of an eHealth app would be that monitoring could provide insight into the course of symptoms, providing information for follow-up visits and receiving personalized advice and tailored supportive care.

Both clinicians and patients were interested in tracking mood, anxiety, and lifestyle behaviors, such as physical activity and diet or nutrition, as areas of shared importance. This may be related to the growing popularity of fitness trackers and health apps in general; nonetheless, the monitoring of physical activity and lifestyle behaviors is of clinical importance, as it strongly reflects the functional status. Furthermore, the fact that patients are interested in monitoring these aspects of their function points to a higher likelihood of adherence when asked to monitor their behaviors. A previously conducted randomized clinical trial tested a system similar to COMPASS in patients with type II diabetes; in that study, the intervention group supported with 24-hour access to mobile health coaching, monitoring, and communication obtained better disease control in half the time that it took the comparison group [35]. This finding highlights the potential benefits of more regular contact that can be facilitated with a system such as COMPASS. The growing interest in eHealth-based approaches in the clinical care setting seemingly reflects the benefits that such systems can provide for both patients and providers.

Other themes representing actionable data included the monitoring of the functional status and the ability of clinicians to communicate the results of tests and imaging with their patients more readily—something that really tapped into the role that innovative systems such as COMPASS could play in ongoing patient care. However, as was mentioned several times, too much data of no relevance to the clinical care of patients would potentially create more of a burden than a benefit. Kuijpers et al reached a similar conclusion when evaluating a Web-based intervention focused on patient care for lung and breast cancer survivors in the Netherlands [36]. While health care professionals supported access to the electronic medical record for providing reports and results, they also expected it would lead to the increased workload because patients were unlikely to understand the information provided, prompting greater burden on clinicians to follow-up. This has important implications for the development and potential of similar eHealth-based approaches as the goal is to provide improved patient care, but not lead to a greater workload, which is already a burden on the system [27].

### Limitations

Because this study was designed to assess user needs relating to an eHealth-based system for monitoring and facilitating communication between patients and clinicians, we could not test the prototype with a large number of patients. The study included only 3 patients with 2 types of diagnoses compared with what would typically be seen in the clinical setting. Therefore, a primary limitation of this study was the small number of patient interviews conducted in phase I of the study, which may somewhat limit any generalizability of our findings to other patient subsets. To address this limitation and facilitate a continuing update of system features and further refine the technology, patients who are involved in phase II will provide regular feedback on usability and features, which will be incorporated with those provided in phase I. Evidence suggests that between 6 and 12 participants can provide adequate data for determining meta-themes [37]. Given the fast pace of obsolescence in technology settings, the priority of phase I of the study was to develop a prototype of the device-agnostic platform that incorporated evidence-based features identified by clinicians and that could be more rigorously tested in phase II. Another limitation is that while the device was given to participants who had previous experience with a smartphone for logistical purposes, this may not reflect well on the ease of adoption for a smartphone-naïve individual.

### Strengths

It is critical that people engaging with the technology—in this case, clinicians, patients, and researchers—have input as to the design of the tools and content they will be using. A significant strength of this study is that we used an iterative process that involved input from researchers, engineers, clinicians, and patients in identifying the aspects of a mobile and eHealth-based platform that would be most effective for monitoring, capturing, and reporting of relevant data related to patient care in the time between standard clinic visits. Other significant strengths of the study were as follows: (1) the use of qualitative methods that allowed us to gather and synthesize provider and patient perspectives “in their own words” and (2) the broad cross-section of clinicians that provided a detailed description of the types of metrics deemed most important and suitable for monitoring and the types of patients who could benefit most from a system like COMPASS.

### Conclusions

Technology is being increasingly integrated into the care of cancer patients and survivors, who as a group require significant resources in terms of time and personal contact. One of the primary goals of technology-driven approaches is to help improve the efficiency of the current care delivery system while providing high-quality care. Technology needs to support the decision-making process of providers in an evidence-based manner and in a way that makes its use easy for patients to comply. It is critical to include the perspective of clinicians and their patients, as well as researchers and engineers, when designing such systems. Furthermore, to provide a continual refinement of such technologies, it is critical that ongoing feedback be sought from patients and clinicians using the system. Many practical and logistical issues may only arise after ongoing use and in response to changing environmental conditions. Therefore, a degree of flexibility in design iteration is preferable. However, the other side of this argument is that asking patients to utilize systems that are burdensome will lead to low compliance while providing clinicians with vast amounts of unusable data will lead to similar results. An ideal system would streamline patient-provider interactions while also highlighting clinically relevant domains that are important to clinicians and ensure patients most salient concerns are addressed. Future research needs to determine whether such systems can be integrated into current practice settings and whether there are, in fact, improvements in care and outcomes for a variety of different cancer patients and survivors.

## Appendix

Multimedia Appendix 1 Thought exercise.

Multimedia Appendix 2 Clinician and patient interview quotes.

Multimedia Appendix 3 Potential sensor list.

## Abbreviations

COMPASS: Capturing and Analyzing Sensor and Self-Report Data for Clinicians and Researchers
eHealth: electronic health
PC: personal computer
PRO: patient-reported outcome
RHLCCC: Robert H Lurie Comprehensive Cancer Center
TCA: thematic content analysis
