# Supplementary material for: Development of an eHealth System to Capture and Analyze Patient Sensor and Self-Report Data: Mixed-Methods Assessment of Potential Applications to Improve Cancer Care Delivery
Source: JMIR Med Inform. 2018 Oct 22;6(4):e46. doi: 10.2196/medinform.9525 (PMC6234343; doi:10.2196/medinform.9525)
Supplement: Multimedia Appendix 1 [file medinform_v6i4e46_app1.pdf]

Thank you for agreeing to participate in our research study “COMPASS: Capturing and Analyzing Sensor and Self-Report Data for Clinicians and Researchers!” In preparation for our 1-hour interview we’d like to provide you with a bit more background so that you can begin thinking about how COMPASS may best benefit your clinical practice as well as patient outcomes.

The purpose of this study is to develop COMPASS, a device agnostic mobile phone and e-tablet compatible platform, that can help clinicians remotely manage patient care in-between medical visits. COMPASS can use patient self-report data- and wearable sensors- to gauge, sample, analyze and securely communicate information about physiology (e.g., temperature, heart rate variability, BP), physical and neurological health (e.g., BMI, gait, balance), behavior (e.g., exercise), and psychosocial wellbeing (e.g., anxiety, depression). Such actionable data can inform real-time revisions to goals of care. Moreover, COMPASS can be used to deliver automated, evidence-based, custom rehabilitation services (e.g., reminders to engage in physical exercise) to cancer survivors.

Given these capabilities, it would be helpful as you’re in clinic to begin considering how such a tool could best improve your efficiency of care and clinical outcomes. If there was a system that could monitor your patient’s in-between clinic visits, report back to you with a summary of how your patients are doing, and alert you if there is something concerning that’s going on with the patient (for example, a high fever or a pain crisis), what would the system look like? Would it monitor all patients or only more complex patients? What specifically would it monitor? In an ideal system how would you receive the information?

Leading up to our interview you might reflect on which potential uses for COMPASS could be the most valuable:

A. Monitoring health and well-being to allow for the detection of abnormalities (e.g., functional impairments necessitating rehabilitation)

B. Measuring adherence to prescribed treatment (e.g., medication usage via electronic medication blister packs) or progress towards goal attainment (e.g., daily hours of mobility, improved nutritional intake, re-engagement with vocation)

C. Identifying the actionable effects of drug therapy (e.g., symptom management, dose delay)

D. Promoting healthy behaviors or enhanced quality of life (e.g., delivering tailored, automated interventions to help survivors monitor and increase physical activity or decrease fear of recurrence)

Bringing your ideas on the aforementioned to the interview will help us maximize our time together. If you have any additional questions in the interim, please do not hesitate to contact us!
