# Supplementary material for: Development of an eHealth System to Capture and Analyze Patient Sensor and Self-Report Data: Mixed-Methods Assessment of Potential Applications to Improve Cancer Care Delivery
Source: JMIR Med Inform. 2018 Oct 22;6(4):e46. doi: 10.2196/medinform.9525 (PMC6234343; doi:10.2196/medinform.9525)
Supplement: Multimedia Appendix 2 [file medinform_v6i4e46_app2.pdf]

Clinician Interviews	
**Theme**	**Quote**
**Priority areas to assess**<br><br>- And, importance of not collecting data that was unactionable	*"The symptoms I feel like we would be alerted to would be decreased appetite, although it's hard to do something about those symptoms, so I think the interventionable … – the things that are actionable would be pain and nausea, although we'd be alerted to other symptoms, they would be unactionable."* [Physician, GI cancers]
**Patients most appropriate for COMPASS**<br><br>- Older patients<br><br>- Patients with complicated treatment regimens	*"with any type of cancer, the older the patient probably the more at risk they are because of frailty and comorbidities."* [Physical Therapist, breast cancer patients]<br><br>*"The regimen's complicated—it's something like four to six pills twice a day and it's only given Monday through Friday. So, they would benefit from something like a compliance check."* [Physician, GI cancers]
**Summarizing patient data collected through COMPASS**<br><br>- Where to view data report	*"To have COMPASS be truly device agnostic would be optimal and allow for checking the data from different locations."* [Physician, neuro-oncology]<br><br>*"Within cancer, it's so multi-disciplinary… and so often nobody talks to anybody about this. So yeah, I would say to share is huge in importance. That would be a major contribution."* [Clinical psychologist, head and neck cancers]
**Patient interviews**	
**Wearability**<br><br>- wrist watch type device	*"…there was also a band to wear around it, and it was too big…The wrist may have not have been great all the time, just depending on the IVs."*<br><br>*"…it wasn't a big device, but I already wear a watch and so to wear something else was different. I understand why you have to wear it tightly but it wasn't so comfortable."*
**Data Capture**<br><br>- Syncing smartphone and wrist watch type device	*"It was difficult to know the range; how close did I have to be to the phone to make sure that they were talking to one another? It would be nice if the light turned red or something when they weren't connected"*

**Tracking**<br><br>- Activity type and why<br><br>- Freehand notation	*"I think it'd be really helpful for an app to be able to do things like track running or also why people do yoga or meditation…showing that you're taking an active role in your health."*<br><br>*"On there, it would be nice to be able to say—angry …. Then again, maybe also adding the freehand angry. Then you could say why"*

Note: We do not identify the patient who provided the quote as is done with clinicians to protect privacy given the small number of patients.
