# Supplementary material for: Development of an eHealth System to Capture and Analyze Patient Sensor and Self-Report Data: Mixed-Methods Assessment of Potential Applications to Improve Cancer Care Delivery
Source: JMIR Med Inform. 2018 Oct 22;6(4):e46. doi: 10.2196/medinform.9525 (PMC6234343; doi:10.2196/medinform.9525)
Supplement: Multimedia Appendix 3 [file medinform_v6i4e46_app3.pdf]

Accelerometer

Gait

Gyroscope

GPS

Indoor Positioning

ECG

Temperature

Heart Rate

Oximeter

Indirect Calorimetry

Electrodermal Response

Heat Flux

Motion

Medication Adherence

Asthma Device Use

Blood Glucose Monitor

Sleep

Weight

Mood
